# Improvement in the Quantification of Foreign Object Defects in Carbon Fiber Laminates Using Immersion Pulse-Echo Ultrasound

**DOI:** 10.3390/ma14112919

**Published:** 2021-05-28

**Authors:** Nathaniel J. Blackman, David A. Jack, Benjamin M. Blandford

**Affiliations:** Department of Mechanical Engineering, Baylor University, Waco, TX 76798-7356, USA; Nathaniel_Blackman@baylor.edu (N.J.B.); Ben_Blandford@baylor.edu (B.M.B.)

**Keywords:** laminates, carbon fiber, foreign objects, ultrasound, non-destructive testing, manufacturing defects

## Abstract

This research presents a new technique using pulse echo ultrasound for sizing foreign objects within carbon fiber laminates. Carbon fiber laminates are becoming increasingly popular in a wide variety of industries for their desirable properties. It is not uncommon for manufacturing defects to occur within a carbon fiber laminates, causing waste, either in the discarding of failed parts or the overdesign of the initial part to account for these anticipated and undetected errors. One such manufacturing defect is the occurrence of a foreign object within the laminate. This defect will lead to a localized weakness within the laminate including, but not limited to, stress risers, delamination, and catastrophic failure. This paper presents a method to analyze high-resolution c-scan full waveform captured data to automatically capture the geometry of the foreign object with minimal user inputs without a-priori knowledge of the shape of the defect. This paper analyzes twelve samples, each a twelve-lamina carbon fiber laminate. Foreign objects are made from polytetrafluoroethylene (PTFE) measuring 0.05 mm (0.002 in.) thick and ranging in diameter from 12.7 mm (0.5 in) to 1.588 mm (0.0625 in), are placed within the laminates during fabrication at varying depths. The samples are analyzed with a custom high-resolution c-scan system and smoothing, and edge detection methods are applied to the collected c-scan data. Results are presented on the sizing of the foreign objects with an average error of 6% of the true area, and an average absolute difference in the estimation of the diameter of 0.1 mm (0.004 in), an improvement over recently presented ultrasonic methods by a factor of three.

## 1. Introduction

Carbon fiber composites are highly desirable due to their high strength to weight ratio and their anisotropy, which allows for parts to be designed with a directionally biased strength. This contrasts with traditional materials, such as metals, that are isotropic in nature with no preferential directionality [1]. Many industries have seen a dramatic increase in the use of composites. The aerospace industry, in particular, has greatly expanded its use of composites [2]. One notable example is the Boeing 787 that is 50 percent composite by weight, with a majority of the primary structure being made up of composite materials [3].

The manufacturing process for composite laminate materials is often a manual process and the composite is susceptible to varying defects. Delaminations, broken fibers, inclusions, incorrect layup, missing layers, etc., are possible defects that are often introduced during manufacturing [2]. These defects are known to reduce the service life of components, acting as stress concentration points in the composite [4]. Carraro et al. [5] observed a reduction in tensile strength due to the existence of voids and used FEA to relate the local shape of a void to the local strain concentration. Wang et al. [6] used micro-CT and modeling to characterize void content in CFRPs and reported a reduction in properties due to these defects.

The need for safer composite parts as well as other engineering and cost demands brings about the need for non-destructive techniques to identify these defects [2]. As of 2018, market research estimates the non-destructive testing and inspection sector is $8.3 billion in size and is projected to grow to $12.6 billion by 2024 [7]. This explosion in value can, in part, be traced to the greater adoption of advanced materials requiring less destructive testing and more non-destructive methods due to the costs of parts as well as government mandated safety checks in industries like aerospace which require substantial man-hours [8].

Several nondestructive techniques have been investigated for their ability to identify foreign objects and delaminations in laminated composites including shearography, thermography, X-ray CT, acoustography, terahertz, and ultrasound [9,10,11,12,13]. Ultrasound is the most popular non-destructive technique [7] and is particularly useful due to its safety, portability, relatively low-cost, and ease of use [2].

Benammar et al. [14] studied the use of signal processing techniques to aid in the detection of delaminations in carbon fiber reinforced composites using ultrasound. They were able to detect the depth of the defects within 4% of the actual depth of the defect just past the 1st lamina, and a max error of 8% for the depth of defects near the back wall. Poudel et al. [15] presented a method for identifying foreign objects in carbon fiber laminates using fuzzy logic an artificial neural network (ANN) to detect Teflon inclusions. They present results for a CFRP panel with twelve Teflon defects where their ANN correctly identifies the existence and shape of the foreign objects, but the accuracy in sizing the foreign objects is not provided. Hasiotis et al. [16] aimed to trace the shape of foreign objects within both glass fiber and carbon fiber laminates using ultrasound c-scan data. The shape of the defects was able to be identified, but size was significantly overestimated in the carbon fiber laminates.

Li et al. [17] demonstrated the ability of an edge detection method based on the standard deviations of the ultrasound data to outline simulated delamination defects created through impact damage. It is unclear what the expected accuracy of the method would be as no quantitative results are provided. Wroknowicz-Katunin et al. [18] researched the capabilities of different image processing techniques to identify BVID in a CFRP panel. The edge detection techniques they tested were found to not be suitable for the analysis of the BVID damage. To the best of the authors knowledge, no substantial research has been conducted on the use of edge detection techniques for the case of manufactured defects.

Work by Amenabar et al. [9] compared the use of shearography, thermography, ultrasonics, and X-ray CT in identifying five Teflon inclusions, which were used to simulate delaminations, within two eight-lamina glass fiber laminated composites. Only ultrasound and thermography successfully detected all five defects in both composites. Ultrasound was reported as having an average lateral error in detecting the area of 15% for a 30 mm circular defect, this average lateral error decreased to 6% for the thermography measurements. Using this error in area, it can be approximated that the diameter of the 30 mm circle was measured to be approximately 32.1 mm in diameter with their techniques or an approximate error of 2 mm in measuring diameter. The size of these inclusions is significantly greater than those used in this work, and the desire to reduce the error well below 2 mm (0.079 in) in a critical dimension is advanced in the present paper.

Poudel et al. [10] compare the abilities of through-transmission acoustography with through-transmission ultrasound and infrared thermography to detect inclusions within a carbon fiber laminate. They fabricated three panels with Teflon inserts of differing shapes and sizes, and placed at different thicknesses throughout the part. The error in the measurement of the diameters for the three 20 mm inclusions at various depths was presented for each of the techniques, and the results indicated a maximum error of 0.4 mm for acoustography, while through-transmission ultrasound had a maximum error of 1.2 mm. The present work is distinct in both its measurement techniques and the use of pulse-echo ultrasound which only requires access to one-side of component.

In another work, Poudel et al. [19] use ultrasound to identify foreign objects, impact damage, and porosity. An artificial neural network is used to analyze a-scans using a nearest mean technique. The authors reported that this method is capable of correctly identifying 98% of the investigated a-scans and the technique is capable of being modified to identify other types of defects, but results were not presented to study or quantify the geometry of the defect.

Barry et al. [20] developed a method for detecting defects using ultrasonic data analyzed by an artificial neural network. The researchers tested the ability of several different algorithms to detect two different types of inclusions, Mylar and Teflon, inside of carbon fiber laminates. The Mylar is considered a bonding defect, while the Teflon is a non-bonding defect meant to simulate a delamination. They were able to take an initial training set and achieved a high level of success classifying a-scans from laminates of varying sizes as being defect free or containing a bonding or non-bonding defect. It was not within the scope of the work of Barry et al. to discuss the quantification of the defects, so it is unknown how well the inclusions were sized.

Mohammadkhani et al. [21] presented an algorithm based around the wavelet transform that is meant to detect and characterize defects. In their work, they analyze ultrasonic data from 5 MHz and 10 MHz phased array transducers. They prefer the better axial resolution that is promised by the 10 MHz phased array elements and address some of the challenges that are inherit in scanning a carbon fiber laminate with higher frequency transducers. They present the results of their algorithm on a twelve-lamina unidirectional carbon fiber panel made in an autoclave with 5 different types of inclusions; Teflon, paper, release tape bag tape, and peel ply; placed between the 8th and 9th lamina and all 6 mm × 6 mm squares. Their algorithm had an estimated error of 2% when predicting the correct depth of the Teflon and a maximum error of 6.8% in predicting the depth of any inclusion. The algorithm overpredicted the size of the Teflon inclusion by 43.8% and a manual estimate of the size underpredicted by 17%.

In a similar work, Ma et al. [22] proposed a signal correlation algorithm for the detection of defects. They tested their algorithm on a carbon fiber laminate with 14 unidirectional laminae, that was made with intentional manufacturing defects to simulate a delamination. The authors in [22] studied three defects, all circular and measuring 12.7 mm (0.5 in) in diameter and 0.25 mm (0.01 in) in thickness were placed in the laminate between the 3rd and 4th laminae, 6th and 7th lamina, and 9th and 10th lamina, respectively. They chose to benchmark their algorithm against ultrasound phased array scans. Their algorithm was less accurate in predicting the depth or area of the defect between the 3rd and 4th lamina. When determining size, the error of their algorithm ranged in error of predicting the height and width from 0.475 mm (0.019 in) to 0.175 mm (0.007 in). 

The work presented in the current paper compliments the works of Mohammadkhani et al. and Ma et al. in [21,22] as it provides a tool for high resolution sizing of a defect but does not seek to solve the problem of precisely identifying the depth of defects. Many of the results in the present paper for the error analysis will be benchmarked against the results presented in [21,22]. The prime differentiator in the application of the present work is in focusing on a woven composite instead of a pristine highly densified unidirectional composite. The material systems of [21,22] avoid the multitude of time varying reflections inherent to a woven laminate system caused by the undulation of the carbon fiber tows within the weave. The research described in this present paper uses a custom ultrasonic c-scan immersion system to inspect carbon fiber laminates for the identification and quantification of foreign object defects from the manufacturing process. Averaging techniques based on a normal distribution and interpolation techniques using the Fourier Transform are shown to improve the quality of a c-scan image. In addition, this study demonstrates the use of the magnitude of the gradient as an edge-finding technique as a viable tool for the sizing of defects. All foreign objects were measured optically prior to the manufacturing process. All samples were scanned using a 7.5 MHz spherically focused Olympus transducer. Results show an average error in determining the diameter of a foreign object of 0.11 mm (0.004 in). This shows an improvement over results published in the literature which range in error from 0.175 mm [22] to 2.1 mm [9] for objects of comparable sizes. In addition, the present work shows success in identifying defects smaller than those previously published. Future research is planned to automate the analysis technique presented, as well as study new types of manufactured defects including irregularly shaped defects. 

## 2. Materials and Methods

### 2.1. Foreign Object Fabrication

In order to facilitate a robust study, twelve Polytetrafluoroethylene (PTFE) foreign objects were fabricated using a Silhouette Cameo. A strip of PTFE measuring 0.05 mm (0.002″) thick was placed on a cutting mat, and the PTFE was cut into twelve circles; three with a 12.7 mm (1/2 in.) diameter, three with a 6.35 mm (1/4 in.) diameter, three with a 3.18 mm (1/8 in.) diameter, and three with a 1.59 mm (1/16 in) diameter. Using regularly shaped, circular defects, allows for a simple and straightforward example of the resolution of the analysis technique.

**Figure 1:**
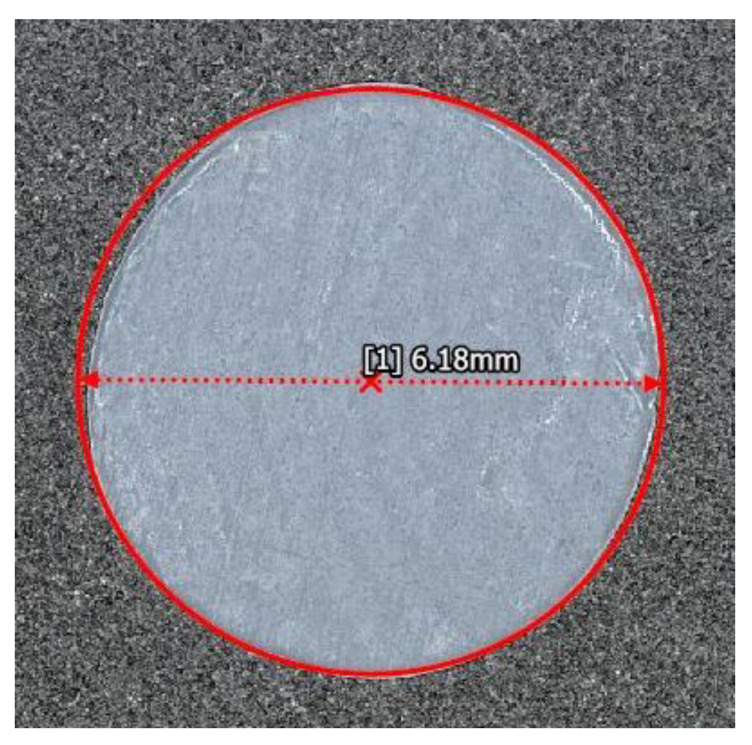
Measurement of the diameter of B2 imaged by the Keyence VR 3000.

The true size of the foreign objects was measured using 3D microscopy (VR-3000, Keyence, Osaka, Japan). The foreign objects have a tendency to curl up on the edges making it difficult to image the cross-section appropriately. In order to combat this, a microscope slide was placed over each foreign object as it was imaged to improve the image quality and accuracy of the sizing technique. Each foreign object was sized at the maximum resolution that would not require the stitching of multiple images. Figure 1 shows an example of the measurement on a piece of PTFE with an intended diameter of 6.35 mm (0.25 in) and is representative of all foreign objects measured. The Keyence’s built-in tool for measuring the effective diameter was used. The VR-3000 Series Software, version 1.4.0.0, requires the user to provide three points along the edge of the foreign object, and the software automatically fits a circle to the imaged object using the software’s built-in edge detection, which utilizes sub pixel processing to determine the edge [23]. To quantify repeatability of the Keyence a manufacture’s provided test slide with known features of 15.000 mm and 5.0000 mm are measured and we obtained values from 20 repeated tests of, respectively, 14.99816±0.00007 mm and 5.00000±0.00003 mm. An additional study was performed on our samples as they produce inconsistent shadowing based upon sample placement that can be misidentified as an edge. For this study we measured two objects with nominal diameters of 6.35 mm (1/4 in.) and 3.18 mm (1/8 in.). For the 6.35 mm feature the standard deviation in repeated measurements was found to be 0.003 mm yielding a typical error of 0.05%, and for the 3.18 mm feature the standard deviation in repeated measurements was found to be 0.21%. Table 1 lists the magnification of each measurement, the measured diameter, and the area of the foreign object. Based upon the measurements taken, the 12.7 mm objects had a manufactured diameter of 12.59 ± 0.07 mm, the 6.35 mm objects had a manufactured diameter of 6.12 ± 0.05 mm, the 3.18 mm objects had a manufactured diameter of 3.11 ± 0.02 mm, and the 1.59 mm objects had a manufactured diameter of 1.55 ± 0.05 mm. It is noteworthy that all fabricated samples are smaller than their nominal sizes which highlights the importance of determining the true sizes of the Teflon foreign objects prior to placement withing the carbon fiber laminate. 

### 2.2. Laminate Fabricaton

Twelve carbon fiber composites were fabricated with a layup of (0/30/60/0/45/0)_s_. During the layup process, intentional PTFE foreign objects were placed between layers 3 and 4, 6 and 7, and 9 and 10 with layer 1 being the layer closest to the tool side. The PTFE samples have an estimated (see e.g., [24]) speed of sound of 1390 m/s and a density of 2140 kg/m^3^, yielding an acoustic impedance of $2.97\times 10^{6}$ Pa-s/m^3^. These foreign objects were of nominal sizes 12.7 mm (1/2 in), 6.35 mm (1/4 in), 3.18 mm (1/8 in), and 1.59 mm (1/16 in), as described above. Only one foreign object was placed within each laminate. The laminates were labeled A–D, according to the size of the foreign object placed in the sample with A corresponding to the largest, 12.7 mm, inclusion and D referring to the smallest, 1.59 mm, inclusion. Each sample was also assigned a number code, 1–3, with code 1 corresponding to a foreign object placed between the third and fourth lamina, code 2 corresponding to a foreign object placed between the sixth and seventh lamina, and code 3 corresponding to a foreign object placed between the ninth and tenth lamina. For example, the sample referred to as A1 had a foreign object with a 12.7 mm (0.5 in) nominal diameter and was placed between the 3rd and 4th lamina from the tool side.

Each laminate was fabricated using 3K, 6 oz. plain weave carbon fiber from ACP composites. The Vacuum Assisted Resin Transfer Method (VARTM) was used to infuse a Proset INF 114 resin and Proset 211 hardener mixture. Each laminate was cured according to the manufacturer’s recommended cure cycle for best properties. After the completion of the cure cycle, a tile saw equipped with a diamond tipped blade was used to remove scrap material. All twelve samples were nominally 50.8 mm × 50.8 mm (2 in × 2 in) after the removal of scrap material. The density of the samples was measured to be 1370 kg/m^3^ with a speed of sound of 2890 m/s, yielding an acoustic impedance of $3.96\times 10^{6}$ Pa-s/m^3^. Of note is that although the acoustic impedance mismatch between the Teflon and the carbon fiber laminate is relatively high ($Z_{PTFE}\sim 3\times 10^{6}$, whereas $Z_{CF}\sim 4\times 10^{6}$), the uniqueness of this configuration is the relative thinness of the PTFE inset at 0.05 mm, whereas the wavelength of the acoustic wave of the 7.5 MHz transducer is effectively 0.37 mm in the carbon fiber and 0.19 mm in the PTFE. 

### 2.3. Scanning Setup

**Figure 2:**
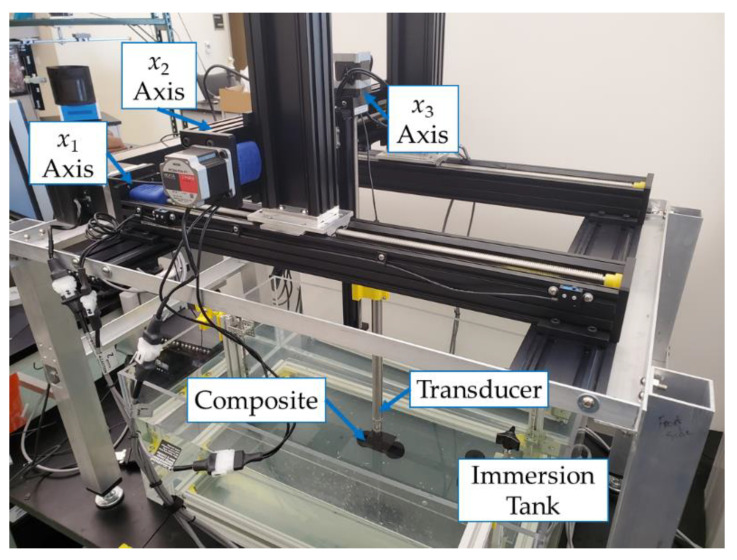
Custom c-scan immersion system used in the present study.

All twelve samples were scanned from the tool-side only using a custom ultrasonic immersion c-scan system as can be seen in Figure 2. Each scan was taken over an area of 38.1 mm × 38.1 mm (1.5 in × 1.5 in) in a raster pattern with a spacing of 0.2 mm (0.008 in) between scans. At the beginning of each scan, the bottom left edge of the sample is found by moving the transducer off the edge of the sample in both the $x_{1}$ and $x_{2}$ dimensions and each scan is started from that location. Before scanning, each sample was leveled to keep the part in focus over the entire range of the scan and ensure the transducer was normal to the surface of the sample.

Lateral resolution is directly related to the frequency of the transducer and can be represented as the minimum distance required to differentiate between two side-by-side structures [25]. The beam width of a transducer, b, can be found as

(1) $$b=\frac{1.4\;\lambda \;f}{D}$$

where λ is the wavelength, f is the focal length, and D is the transducer diameter. Equation (1) suggests that increasing the frequency of the transducer will improve the spatial resolution. Each sample was scanned using a 9.53 mm (3/8 in) spherically focused 7.5 MHz Videoscan transducer (V320-SU-F1.50IN-PTF, Olympus, Tokyo, Japan) with a nominal focal length of 38.1 mm (1.5 in) with a peak frequency of 7.71 MHz, center frequency of 7.15 MHz and a −6 dB bandwidth of 76.22%. Internal studies have shown transducers up to 15 MHz capable of detecting foreign objects in carbon fiber laminates. The transducer was focused at the midplane of each sample prior to scanning. An ultrasonic pulser/receiver (EUT 3160, US Ultratek, Walnut Creek, CA, USA) is used to drive the transducer with a 65 ns negative square wave pulse width at 200 V, and data was captured at a rate of 160 MHz. The transducer is moved by two Velmex BiSlide translation stages. Both the $x_{1}$ and $x_{2}$ translation stages have a resolution of 0.005 mm/step. A Linear Voltage Displacement Transducer (LVDT) from RDP Electronics is used to monitor the position of the transducer along the $x_{2}$ dimension. Custom LabVIEW programs were developed in-house and are used to control all components of the c-scan system. Custom MATLAB codes are then used to analyze the ultrasonic data captured during the scanning process.

## 3. Results

### 3.1. A-Scan Preprocessing Methods

**Figure 3:**
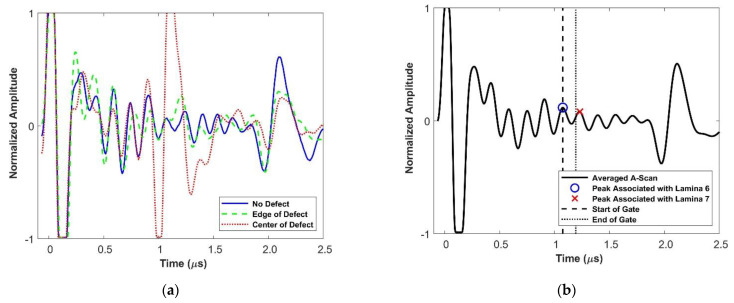
(**a**) Comparison of a-scans over a region with a defect and one that is defect free (**b**) Averaged a-scan used to determine proper depth for the start of a c-scan gate. Average is taken over all a-scans from the sample.

A-scans represent the normalized wave energy as a function of time and are the most basic representation of ultrasonic scan data. Figure 3a shows the ultrasonic response at three different locations within a representative sample: a defect free region, over the center of the foreign object, and near the edge of the foreign object. Figure 3b shows the averaged a-scan for the sample. All four waveforms from Figure 3 show some similar characteristics. They all have a front wall echo that occurs at t=0 and a back-wall echo that occurs at t=2.2 μs. The variations of the waveforms are due to reflections and waveform scattering off of internal phenomena. The periodic aspect of the waveform is sometimes referred to structural noise, which describes noise created within the structure by heterogenous structures, specifically the layered nature of the laminate [26]. Mohammadkhani et al. identified structural noise as a particular challenge in using higher frequency transducers [21]. This issue was due to the increased scattering of the ultrasonic waves causing the signal to attenuate more quickly. In order to combat this, the signal, in the present study, was saturated at the front wall causing clipping in contrast to normal practice. This was done to provide better resolution of the internal features of the samples that is necessary when scanning using a high frequency transducer.

Figure 3a helps to demonstrate the challenges in relying on a-scan data to properly size defects. The a-scan over the center of the foreign object shows a clear and strong ultrasonic response to the defect at t=1.2 μs. The a-scan taken at the edge of the foreign object shows a nominal change from the waveform over a region with no defect at t=1.2 μs. There is the potential that this change would be mistaken as a change in the structural noise not associated with the defect. This makes it particularly challenging to rely solely on a-scans to properly size foreign objects.

For each part, the averaged a-scan is used to identify the peak associated with the lamina at the front of the interface where the foreign object was placed and used as the start of the gate of the c-scan (i.e., for a foreign object between laminae 6 and 7, the peak associated with lamina 6 is identified as the start of the gate and is shown in Figure 3b). The gate is taken over three quarters of the average peak-to-peak distance.

### 3.2. Enhancement of the Ultrasonic Data

It is important to appropriately shift the ultrasonic scan data in time to account for small variations in the surface of the sample or problems with leveling. Properly shifting ultrasonic data of parts with curvature is complex and one approach to shifting parts with curvature is demonstrated by Nelson et al. [27]. However, the samples in this study are known to be flat allowing for a simpler method of shifting the data. The ultrasonic scan intensity, $ℱ\;\left(x_{1,k},x_{2,l},t\right)$, represents the ultrasonic response at the kth location in $x_{1},$ and the lth location in $x_{2}$ at a given time t, where $k=1,2,\ldots N_{1}$, and $l=1,2,\ldots N_{2}$. The ultrasonic scan intensity is interrogated for the first value in time for each spatial location $\left(x_{1,k},x_{2,l}\right)$ above a set detection threshold. Then, the first peak to occur after this value in time is defined as the front wall echo for a given location $\left(x_{1,k},x_{2,l}\right)$. Using these locations in time, a 3rd order polynomial surface in $\left(x_{1},x_{2}\right)$ is fit to the data. The values for each point in space of this 2-dimensional surface are used to shift the scan data at that location such that for all $x_{1}$ and $x_{2}$, the front wall occurs at $t=t_{0}$. We define the shifted time as $\tilde{t}=t-t_{0}$. The shifted ultrasound scan data will be referred to as $F\left(x_{1,k},x_{2,l},\tilde{t}\right)$.

Using maxima from the averaged a-scan, a location relating to the lamina above the foreign object is determined and the signal is gated to cover three quarters of the peak-to-peak distance between laminae. A modified version of the maximum gated amplitude (MGA) method presented by the authors in [28] is used to determine the value of each $\left(x_{1},x_{2}\right)$ location and is described as:

(2) $$\hat{F}\left(x_{1,k},x_{2,l}\right)=\underset{\tilde{t}\in {\tilde{t}}_{n},{\tilde{t}}_{n+Q}}{\max}(F\left(x_{1,k},x_{2,l},\tilde{t}\right)$$

In the previous work, the value at each location in the gate was found by taken the maximum amplitude of the absolute signal. It was found that not using the absolute of the signal yielded a preferable visual result. The value of Q was set at three quarters of the peak-to-peak distance between plies.

**Figure 4:**
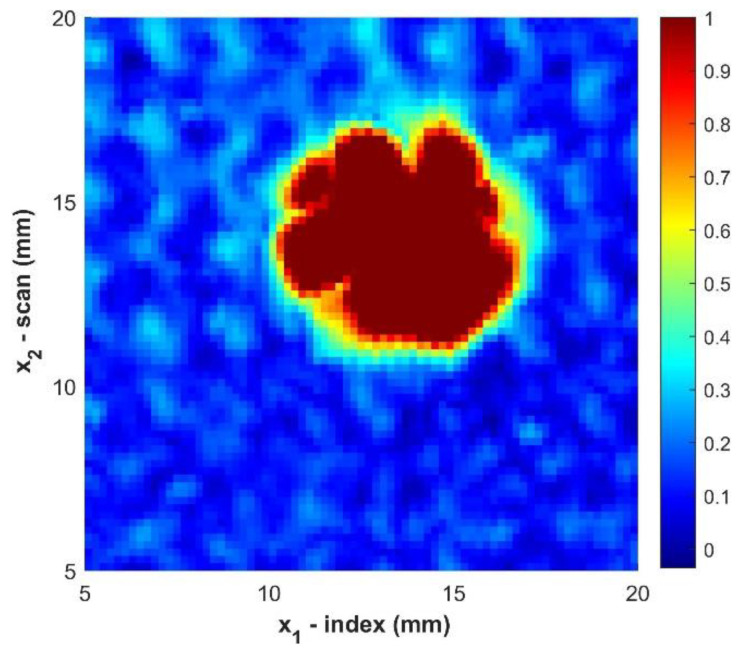
C-Scan of sample B2 prior to any filtering. Color indicates the normalized amplitude of the ultrasonic data.

Using the MGA approach, a c-scan image of the defect can be created as shown in Figure 4. The size and apparent shape of the defect can be observed. However, the edges of the boundary appear grainy due to the limited resolution of the scan and the noisy nature of the material.

In order to further improve the scan data a two dimensional gaussian filter, similar to the one dimensional filter described in [29], was applied across the data as:

(3) $$P\left(x_{1},x_{2},\mu_{1},\mu_{2},\sigma_{1},\sigma_{2}\right)=\frac{1}{2\pi \sigma_{1}\sigma_{2}}\left(e^{-\frac{{\left(x_{1}-\mu_{1}\right)}^{2}}{2\sigma_{1}^{2}}}\right)\left(e^{-\frac{{\left(x_{2}-\mu_{2}\right)}^{2}}{2\sigma_{2}^{2}}}\right)$$

where $\mu_{1}$ and $\mu_{2}$ are the centroidal location of the filter and $\sigma_{1}$ and $\sigma_{2}$ are the standard deviation of the filter (i.e., the spread). Notice that $\int_{-\infty }^{\infty }\int_{-\infty }^{\infty }P\left(x_{1},x_{2},\mu_{1},\mu_{2},\sigma_{1},\sigma_{2}\right)dx_{1}dx_{2}$ is normalized and equals unity. The shifted ultrasound data is then smoothed at each location $\left(x_{1,k},x_{2,l}\right)$ by multiplying the shifted ultrasound scan data by the filter and integrating over all space as:

(4) $$\tilde{F}\left(x_{1,k},x_{2,l},t\right)=\frac{1}{2\pi \sigma_{1}\sigma_{2}}\overset{\infty }{\underset{-\infty }{\int }}\overset{\infty }{\underset{-\infty }{\int }}e^{\frac{-{\left({\tilde{x}}_{1}-x_{1,k}\right)}^{2}}{2\sigma_{x_{1}}^{2}\;}}e^{-\frac{{\left({\tilde{x}}_{2}-x_{2,l}\right)}^{2}}{2\sigma_{x_{2}}^{2}}}\hat{F}\left({\tilde{x}}_{1},{\tilde{x}}_{2},t\right)d{\tilde{x}}_{1}d{\tilde{x}}_{2}$$

Notice in Equation (4) that the variables of integration are ${\tilde{x}}_{1}$ and ${\tilde{x}}_{2}$ and the index locations $\left(x_{1,k},x_{2,l}\right)$ are the locations of the centroid for the filter. Equation (4) is evaluated numerically using the trapezoid rule over the discrete data as:

(5) $$\tilde{F}\left(x_{1,k},x_{2,l},t\right)\approx \frac{\Delta x_{1}\Delta x_{2}}{2\pi \sigma_{1}\sigma_{2}}\overset{m}{\underset{p=-m}{\sum }}\overset{n}{\underset{q=-n}{\sum }}e^{\frac{-{\left(x_{1,k+p}-x_{1,k}\right)}^{2}}{2\sigma_{x_{1}}^{2}\;}}e^{\frac{{\left(x_{2,l+q}-x_{2,l}\right)}^{2}}{2\sigma_{x_{2}}^{2}}}\hat{F}\left(x_{1,k+p},x_{2l+q},t\right)$$

**Figure 5:**
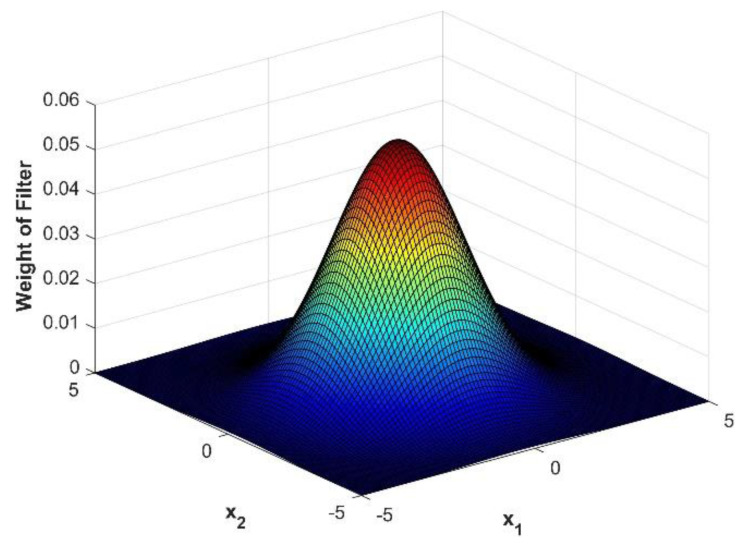
Visual representation of the weights of the gaussian filter with a step size of 5 and standard deviation of 5/3.

**Figure 6:**
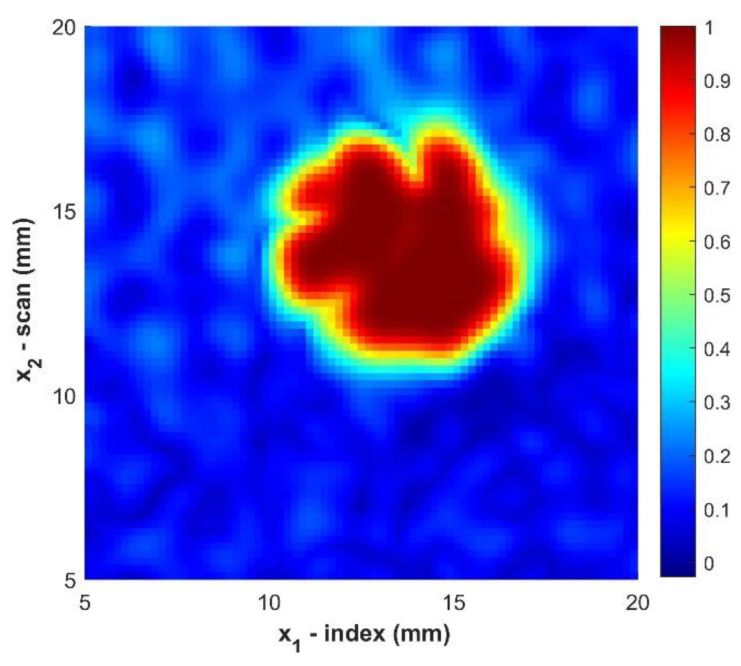
C-Scan of sample B2 after applying a gaussian filter. Color indicates the normalized amplitude of the ultrasonic data.

This filter mutes the effects of spurious intensity that may lead to increased noise when identifying foreign objects and helps to improve the resolution at the edge of the foreign object. For the present work the standard deviation, both $\sigma_{x_{1}}$ and $\sigma_{x_{2}}$, was selected to be 5/3 of the standard step size, and m and n were set to 5 steps. Thus, truncating the integral over all space to a finite region with a loss of less than 0.5% of the full integration. Figure 5 provides a visual of the gaussian distribution for the parameters used and shows that the information near the centroid primarily contributes to the smoothing of the data. Figure 6 shows the improvement of the ultrasonic data after applying the gaussian filter of Equation (5).

Proper smoothing of the data helps to improve the boundaries around the foreign object, but the resolution of the edges of the foreign objects is limited by the resolution of the scan data. The time cost of scanning with a finer resolution, as well as the increased computation costs from the larger data set, make finer scanning resolutions impractical in many cases. Interpolation of the data can be used to solve this issue in the c-scan data. It was determined that interpolating the data after applying the MGA method was preferable and less computationally expensive. It is also important to first smooth the data to prevent the interpolation from propagating numerical noise in the interpolation functions. The process proposed by the authors is to first interpolate $\tilde{F}\left(x_{1,k},x_{2,l}\right)$ along $x_{1}$ using a Fourier transform interpolation to an intermediate dataset $H^{\prime }\left(x_{1,i},x_{2,l}\right)\;$and then use the Fourier transform to interpolate along $x_{2}$ to an upsampled dataset $H\left(x_{1,i},x_{2,j}\right)$. The new upsampled data set uses the indices i and j to replace k and l, as described in Equations (6) and (7). In the present study, the sampling density was increased fivefold in both dimensions for visual purposes. Thus, the indices and data points become:

(6) $$i=1,2,\ldots \;5N_{k};\;x_{1,1}=0\;;x_{1,5N_{k}}=\max\left(x_{1}\right)$$

(7) $$j=1,\;2,\ldots 5N_{l};\;x_{2,1}=0\;;x_{2,5N_{l}}=\max\left(x_{2}\right)$$

**Figure 7:**
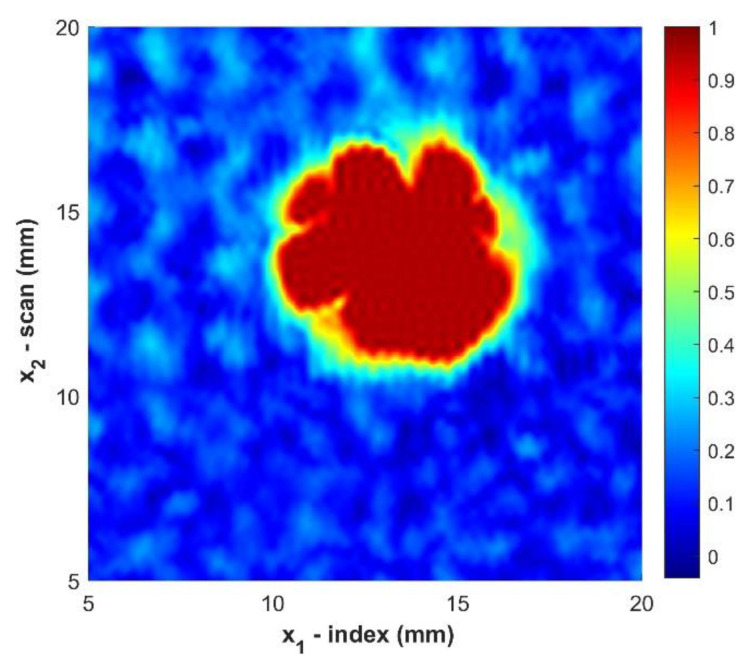
C-Scan of sample B2 using only interpolation. Color indicates the normalized amplitude of the ultrasonic data.

This was implemented in MATLAB using the interpft command [30]. Previous research has shown that interpolation using the Fast Fourier Transform is highly accurate for wavelengths not near the Nyquist limit [31]. Figure 7 shows the improvement in the resolution due to interpolation only of the original data and can be compared to the original image from Figure 4.

**Figure 8:**
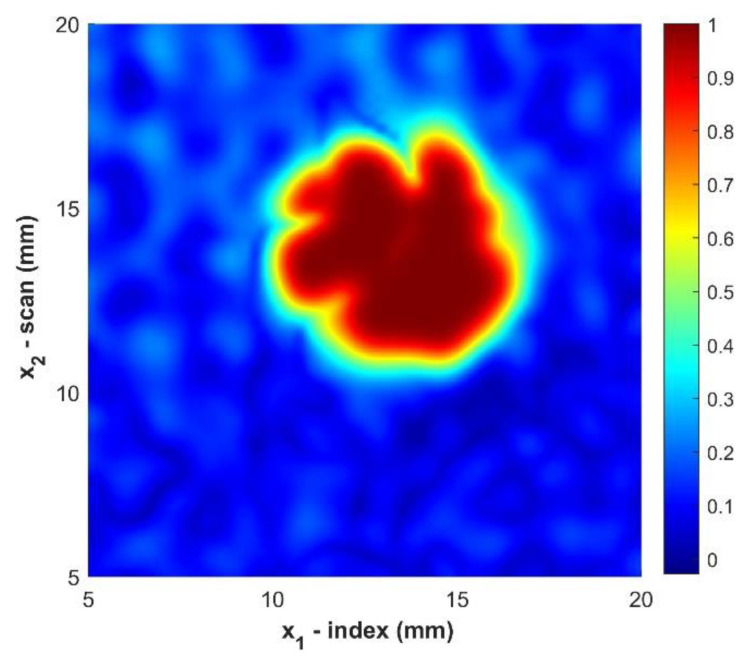
C-Scan of sample B2 after applying both smoothing and interpolation. Color indicates the normalized amplitude of the ultrasonic data.

While Figure 7 does show improvement of the raw c-scan, the edges of the foreign object still exhibit a graininess that is due to the propagation of noise. The data is best improved by first applying the gaussian filter to the data smooth the edges and then interpolate the data to further improve the resolution at the boundary of the foreign object. Figure 8 exhibits the improvement of the image after applying both the gaussian filter and then interpolating the data.

### 3.3. Use of Gradient for Edge Detection

Even with the improvement of the c-scan image it is still difficult to determine the boundary of the inclusion. Edge detection is fundamental to image analysis and simple edge detection can be accomplished using a derivative [32]. The magnitude of the gradient of $H\left(x_{1},x_{2}\right)$ is used in this work to perform a two-dimensional edge detection and is given as

(8) $$G\left(x_{1,i},x_{2,j}\right)=\sqrt{{\left(\frac{\partial H\left(x_{1,i},x_{2,j}\right)}{\partial x_{1}}\right)}^{2}+{\left(\frac{\partial H\left(x_{1,i},x_{2,j}\right)}{\partial x_{2}}\right)}^{2}\;}$$

The c-scan data is discrete, which requires the calculations of the partial derivatives in both dimensions using a numerical method. A $O\left(h^{4}\right)$ central difference method (see e.g., [33]) was chosen for its accuracy and the following equations evaluate the partial derivative Equation (8) as:

(9) $$\frac{\partial H\left(x_{1,i},x_{2,j}\right)}{\partial x_{1}}\approx \frac{-H\left(x_{1,i+2},x_{2,j}\right)+8H\left(x_{1,i+1},x_{2,j}\right)-8H\left(x_{1,i-1},x_{2,j}\right)+H\left(x_{1,i-2},x_{2,j}\right)}{12h}$$

(10) $$\frac{\partial H\left(x_{1,i},x_{2,j}\right)}{\partial x_{2}}\approx \frac{-H\left(x_{1,i},x_{2,j+2}\right)+H\left(x_{1,i},x_{2,j+1}\right)-8H\left(x_{1,i},x_{2,j-1}\right)+H\left(x_{1,i},x_{2,j-2}\right)}{12h}$$

**Figure 9:**
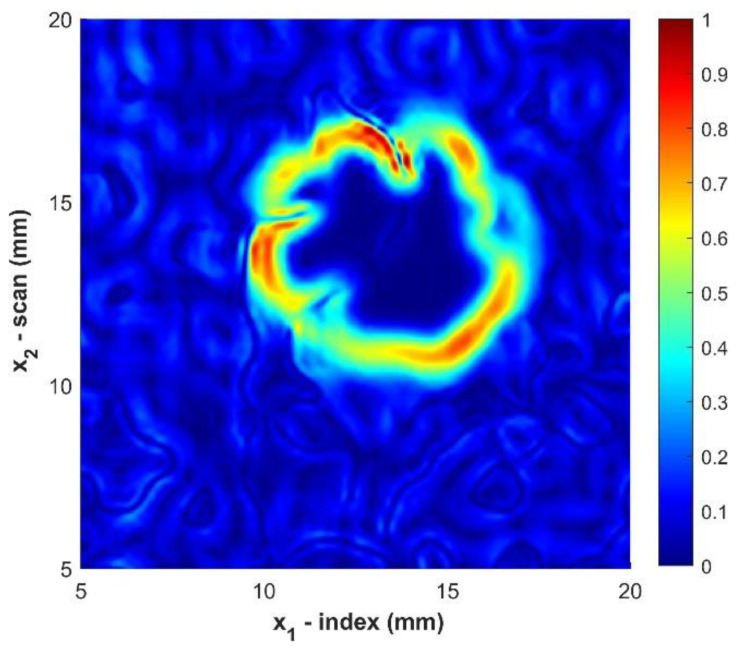
Magnitude of the gradient of the gated c-scan for sample B2. Color indicates the normalize amplitude of the magnitude of the gradient.

Equation (8), using Equations (9) and (10) for the numerical approximations of the gradient, is evaluated at every point $x_{1,i},\;x_{2,j}$. The result for the sample B2 is shown in Figure 9.

**Figure 10:**
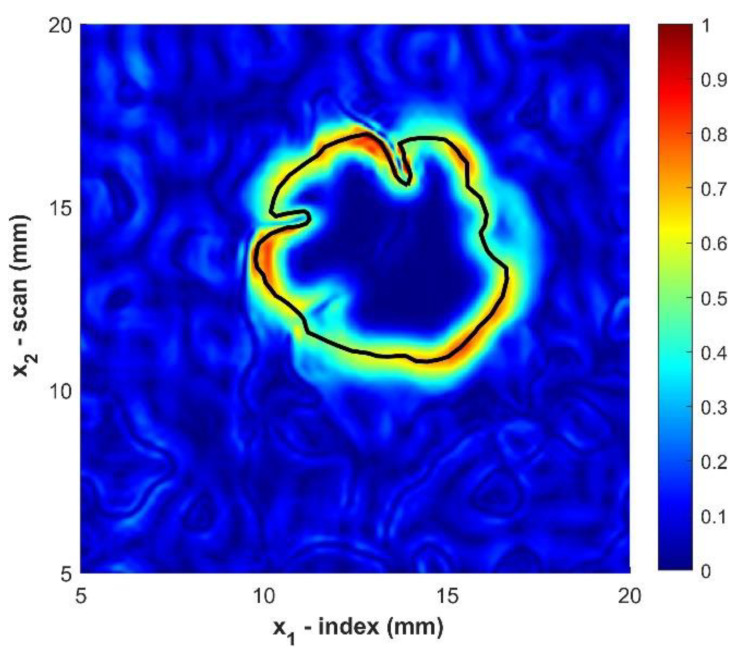
Magnitude of the gradient manually traced for Sample B2. Color indicates normalized amplitude.

The final step in analyzing the size of the foreign object is capturing the peak of the magnitude of the gradient along the boundary of the object. This point will correspond to the greatest signal intensity change and occurs at the transition from the foreign object to the surrounding polymer matrix. This method is termed MGT, maximum gradient transition. The point identified at the outer edge for the foreign object is obtained by first selecting a point manually on the interior boundary. This is any point on the interior surface, and the method was found to be insensitive to any value between 0.1 and 0.3. From this point a projection is formed along the direction of the maximum increase in the gradient. Along this projection the maximum value is selected and tabulated. A new point is then selected on the interior boundary and the process is repeated to capture the peak value along the projection line formed by the direction of the maximum of the magnitude of the gradient. This process continues around the interior boundary of the foreign object region forming the set of points corresponding toe the peak detection on the boundary. In regions where the maximum point is not distinct but there are multiple points due to the limited resolution of the analog to digital converter to capture the acoustic waveform, the center of the set of maximum values is used. It is important to highlight that this method of analysis does not presuppose any given shape. An example of capturing the peak of the gradient that defines the edge of the foreign object is shown in Figure 10 for sample B2. This particular example is selected as it is well below the threshold dimension used in previous studies and, based upon the obtained waveforms, the resulting shape is not that of a circle. It is worthwhile to note that the foreign object is itself of a similar dimension to that of the fiber tows, which may potentially cause a deformation in the foreign object itself. The process described above yields consistent and repeatable results regardless of the location selected within the interior boundary of the magnitude of the gradient plot. Thus, this process can be automated in the future to make the process of sizing defects even more robust. The resulting identified boundary is then post processed in MATLAB to extract the area of the closed surface.

### 3.4. Sizing of Foreign Objects

One of the most common and traditional sizing techniques in the field of ultrasound is the 6 dB drop technique [34,35,36]. This method defines the edge of the defect as point at which the amplitude of the signal is equal to 50% of the amplitude over the defect. In order to demonstrate both the improvement in the c-scan image and the need for a better sizing method the 6 dB method is applied to the c-scan image for the sample B2 as shown in Figure 4, Figure 5, Figure 6 and Figure 7. Table 2 provides the sizing results for sample B2 as well as the average error of the area measurement defined as:

(11) $$\varepsilon =\frac{abs\left(\;A_{True}-A_{MGT}\right)}{A_{True}}$$

where $A_{True}$ is the measured area of the foreign object using the Keyence VR3000 as presented in Table 1. The average error in the area, as a function of size, is larger for smaller foreign objects making it appear that the method is less effective at sizing small foreign objects. To help quantify the resolution of the method an effective diameter, $\bar{d}$, was determined for each foreign object using:

(12) $$\bar{d}=2\sqrt{\frac{A_{MGT}}{\pi }}$$

These effective diameters are used to calculate the absolute difference in the sizing of the diameter for each foreign object.

**Figure 11:**
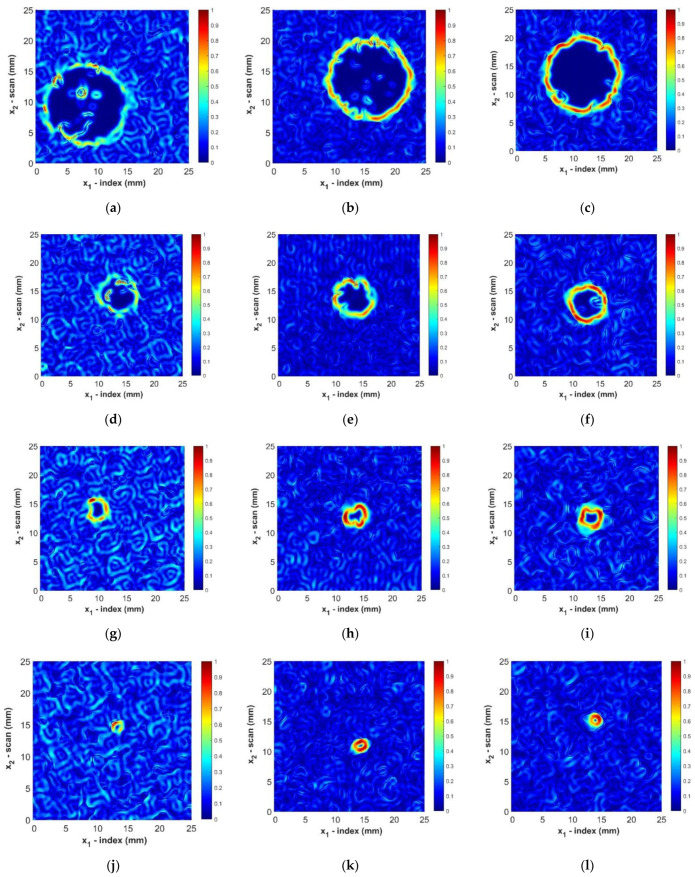
Magnitude of the gradient applied to determine edge of foreign objects, where the color represents the normalized intensity of the gradient: (**a**) Sample A1; (**b**) Sample A2; (**c**) Sample A3; (**d**) Sample B1; (**e**) Sample B2; (**f**) Sample B3; (**g**) Sample C1; (**h**) Sample C2; (**i**) Sample C3; (**j**) Sample D1; (**k**) Sample D2; (**l**) Sample D3.

The results in Table 2 indicate the improvement of the ultrasonic data by the proposed filtering but highlight the need for a better technique for determining the size of foreign objects. The results of sizing foreign objects using the MGT method are shown in Table 3. The MGT method had a maximum error of 0.32 mm (0.013 in) in determining the diameter for all sizes of foreign objects. The average absolute difference in finding the equivalent diameter is 0.11 mm (0.004 in), which is slightly smaller than the resolution of the actual c-scan data itself. The MGT presents a further improvement in the sizing of sample B2 in comparison with the 6 dB drop technique with advanced filtering. Figure 11 presents the magnitude of the gradient for all twelve samples studied. Notice that all defects are clearly visible, with the edges of all defects clearly visible regardless of size or depth within the part.

The average error of the diameter as a function of depth is provided in Table 4. The results indicate no appreciable difference in the accuracy of the measurements of the defects at the 6|7 and 9|10 interfaces, while there is a noticeable increase in error for the foreign objects located at the 3|4 interface. This result agrees with the findings presented in [22], where there was a marked increase in error of the calculated size for the foreign object near the scan surface and more consistent accuracy reported for the foreign objects at greater depths within the part. A possible explanation of this increased error is due to the higher overall signal intensity at this interface location. All scans were performed with the same scanning parameters, simulating a technician with no a priori knowledge of the depth of the foreign objects. It is theorized that reducing the initial gain of the signal for shallow defects during scanning will improve the resolution at the interfaces nearer the scanning surface and is the topic of a future study.

It is helpful to compare the results of the MGT with other recent work in detecting intentional manufactured defects. As noted in Section 1, the results presented [21,22] represent some of the most recent and accurate methods for detecting and sizing defects, but their results were for highly densified unidirectional composites. Their results for that of their unidirectional composites are compared with the results from this paper in Figure 5. for a woven composite system. In order to better compare the results across studies, the difference in characteristic length is presented for all methods. For the circular defects this is the effective area $\bar{d}$ as described in Equation (12), whereas for the square defects this is the length of the side of the square and is defined as s. The absolute difference is estimated for the work of [21] using the presented and measured areas for the Teflon foreign object. The other materials presented in their study are not comparable with the work presented in the current study, although greater accuracy was reported in [21], when measuring Release Tape. In the work of [22], the only material studied is graphite. Table 5. also presents the expected error in the measurement of the area of a foreign object with a diameter of 6 mm for each technique. This is done by calculating the area that would be measured by each method if it had its average error in calculating the diameter. It is important to compare the error for similarly sized foreign objects as the error in measuring area increases as the area of the foreign object decreases assuming the accuracy in determining the diameter is consistent. The MGT method presents a noticeable improvement in the determination of the size of foreign objects, and has nearly three times the accuracy of previously published results (see e.g., [21,22]). It is worth noting that the resulting features are circular in nature but not perfect circles. This irregularity is caused by the variations of acoustic path taken to reach each defect at each $\left(x_{1},x_{2}\right)$ location. Based on CT observations performed in-house, the features themselves are not observed to have a significant out-of-plane deformation, but it is clear from Figure 11 coupled with the known dimensions of the 3K tows that the edge irregularity is on the same length order as the tow spacing itself. This irregularity of the edge will cause subtle acoustic path variations as a function of space and depth that is inherent to woven composites and it would not be expected in a unidirectional laminate.

## 4. Conclusions and Final Remarks

A non-destructive technique using immersion, pulse-echo ultrasound is presented for high accuracy measurement of foreign objects defects within fiber laminates. A simple but robust edge detection method based on the calculation of the gradient was presented to quantify the size of foreign objects. The average error across all foreign objects was 4.9%, but this error increases as the size of the inclusion decreases. A more appropriate quantification is the differential error, and results presented show an error of 0.00 mm (0.000 in) to 0.32 mm (0.013 in) with an average of 0.11 mm (0.004 in) across all foreign objects. Additionally, it was shown that there was not a significant difference in the accuracy of the method for the foreign objects between the 6|7 and 9|10 interfaces and the nominal decrease in accuracy at the 3|4 interface is believed to be caused by holding scan parameters consistent for all scans with no consideration given for the anticipated depth of the foreign objects within the part. These values represent a significant improvement over other recently published works attempting to size Teflon foreign objects in carbon fiber laminates.

This improvement in the detection capabilities of ultrasound could be important in both manufacturing, maintenance, and modeling of components, allowing technicians to better quantify manufacturing defects and engineers to make use of modern computation power and more accurate information of manufacturing defects in determining safety decisions. Future work is planned to investigate the ability of the method to detect manufactured defects of different material, irregular objects, and automate the sizing of the defects.

## Figures and Tables

**Table 1:** Summary of Imaging Results of PTFE Inclusion Using 3D Microscopy.

Foreign Object	Magnification	Diameter (mm)	AREA (mm^2^)
A1	12	12.67	126.08
A2	12	12.55	123.70
A3	12	12.55	123.70
B1	25	6.08	29.03
B2	25	6.18	30.00
B3	25	6.10	29.22
C1	50	3.10	7.55
C2	50	3.13	7.69
C3	50	3.09	7.50
D1	120	1.52	1.81
D2	120	1.61	2.04
D3	120	1.52	1.81

**Table 2:** Impact of filters on sizing of sample B2 using traditional 6 dB drop method.

Filtering Technique	Traced Area (mm^2^)	Absolute Error (%)	Effective Diameter (mm)	Absolute Difference (mm)	Absolute Difference (in)
None	35.24	17	6.70	0.52	0.020
Gaussian Only	35.22	17	6.70	0.52	0.020
Interpolation Only	34.46	15	6.62	0.44	0.017
Both Filters	32.21	7	6.40	0.22	0.009

**Table 3:** Summary of Ultrasonic Measurements using MGT.

Foreign Object	Traced Area (mm^2^)	Absolute Error (%)	Equivalent Diameter (mm)	Absolute Difference (mm)	Absolute Difference (in)
A1	132.51	5.1	12.99	0.32	0.013
A2	127.75	3.3	12.75	0.20	0.008
A3	124.73	0.8	12.60	0.05	0.002
B1	30.81	6.1	6.26	0.18	0.007
B2	30.49	1.6	6.23	0.05	0.002
B3	28.43	2.7	6.02	0.08	0.003
C1	8.41	11.5	3.27	0.17	0.007
C2	7.78	1.2	3.15	0.02	0.001
C3	7.53	0.4	3.10	0.01	0.000
D1	1.82	0.1	1.52	0.00	0.000
D2	1.87	8.1	1.54	0.07	0.003
D3	1.48	18.5	1.37	0.15	0.006
Average	-	4.9	-	0.11	0.004

**Table 4:** Error in diameter as a function of depth using MGT.

Laminar Interface	Average Absolute Difference of Diameter (mm)	Average Absolute Difference of Diameter (in)
3|4	0.17	0.007
6|7	0.08	0.003
9|10	0.07	0.003

**Table 5:** Comparison of MGT method with other Manufactured Defect Sizing Methods.

Method	Absolute Difference of Characteristic Length (mm)	Expected Average Error Percentage 6 mm Diameter Inclusion (%)
6 dB drop with Filtering	$\bar{d}$ = 0.22	7
Wavelet Algorithm [21]	s = 0.52 ^†^	17
Signal Correlation [22]	$\bar{d}$ = 0.29 ^††^	9
MGT	$\bar{d}$ = 0.10	3 *

^†^ The estimated error of the characteristic length using the provided measured and original areas for the Teflon foreign object in [21]. ^††^ The average difference of the provided results in [22]. * The actual average error in measuring the area for the foreign objects (sample code B) with 6 mm diameters in this study.

## Data Availability

The data presented in this study may be made available upon request to corresponding author. The data are not publicly available according to the requests of the research sponsors.
